# The profile of HIV-1 drug resistance in Shanghai, China: a retrospective study from 2017 to 2021

**DOI:** 10.1093/jac/dkad370

**Published:** 2024-02-01

**Authors:** Min Zhang, Yingying Ma, Gang Wang, Zhenyan Wang, Qianying Wang, Xin Li, Feng Lin, Jianping Qiu, Daihong Chen, Yinzhong Shen, Chiyu Zhang, Hongzhou Lu

**Affiliations:** Shanghai Clinical Research Center for Infectious Disease (HIV/AIDS), Shanghai Public Health Clinical Center, Fudan University, Shanghai 201508, China; Shanghai Clinical Research Center for Infectious Disease (HIV/AIDS), Shanghai Public Health Clinical Center, Fudan University, Shanghai 201508, China; Shanghai Clinical Research Center for Infectious Disease (HIV/AIDS), Shanghai Public Health Clinical Center, Fudan University, Shanghai 201508, China; Shanghai Clinical Research Center for Infectious Disease (HIV/AIDS), Shanghai Public Health Clinical Center, Fudan University, Shanghai 201508, China; Shanghai Clinical Research Center for Infectious Disease (HIV/AIDS), Shanghai Public Health Clinical Center, Fudan University, Shanghai 201508, China; Shanghai Clinical Research Center for Infectious Disease (HIV/AIDS), Shanghai Public Health Clinical Center, Fudan University, Shanghai 201508, China; Shanghai Clinical Research Center for Infectious Disease (HIV/AIDS), Shanghai Public Health Clinical Center, Fudan University, Shanghai 201508, China; Shanghai Clinical Research Center for Infectious Disease (HIV/AIDS), Shanghai Public Health Clinical Center, Fudan University, Shanghai 201508, China; Shanghai Clinical Research Center for Infectious Disease (HIV/AIDS), Shanghai Public Health Clinical Center, Fudan University, Shanghai 201508, China; Shanghai Clinical Research Center for Infectious Disease (HIV/AIDS), Shanghai Public Health Clinical Center, Fudan University, Shanghai 201508, China; Shanghai Clinical Research Center for Infectious Disease (HIV/AIDS), Shanghai Public Health Clinical Center, Fudan University, Shanghai 201508, China; Shanghai Clinical Research Center for Infectious Disease (HIV/AIDS), Shanghai Public Health Clinical Center, Fudan University, Shanghai 201508, China; National Clinical Research Center for Infectious Disease, The Third People’s Hospital of Shenzhen, Second Hospital Affiliated to Southern University of Science and Technology, Shenzhen 518112, Guangdong, China

## Abstract

**Background:**

HIV-1 drug resistance is a huge challenge in the era of ART.

**Objectives:**

To investigate the prevalence and characteristics of acquired HIV-1 drug resistance (ADR) in Shanghai, China.

**Methods:**

An epidemiological study was performed among people living with human immunodeficiency virus (PLWH) receiving ART in Shanghai from January 2017 to December 2021. A total of 8669 PLWH were tested for drug resistance by genotypic resistance testing. Drug resistance mutations (DRMs) were identified using the Stanford University HIV Drug Resistance Database program.

**Results:**

Ten HIV-1 subtypes/circulating recombinant forms (CRFs) were identified, mainly including CRF01_AE (46.8%), CRF07_BC (35.7%), B (6.4%), CRF55_01B (2.8%) and CRF08_BC (2.4%). The prevalence of ADR was 48% (389/811). Three NRTI-associated mutations (M184V/I/L, S68G/N/R and K65R/N) and four NNRTI-associated mutations (V179D/E/T/L, K103N/R/S/T, V106M/I/A and G190A/S/T/C/D/E/Q) were the most common DRMs. These DRMs caused high-level resistance to lamivudine, emtricitabine, efavirenz and nevirapine. The DRM profiles appeared to be significantly different among different subtypes.

**Conclusions:**

We revealed HIV-1 subtype characteristics and the DRM profile in Shanghai, which provide crucial guidance for clinical treatment and management of PLWH.

## Introduction

HIV/AIDS is still a major challenge to global public health. During the past 3 years, the COVID-19 epidemic has largely delayed achievement of the goal of global elimination of AIDS as a public health threat by 2030.^1,2^ As of 2022, over 37 million patients are living with HIV globally and about 1.1 million of them are living in China.^3,4^ The Chinese government initiated the national AIDS control policy ‘Four Frees and One Care’ and established the National Free Antiretroviral Treatment Program (NFATP) in 2003. Currently, over 90% of confirmed people living with HIV (PLWH) in China are receiving ART, and the overall mortality of PLWH decreased from 39.3 per 100 person-years in 2002 to 14.2 per 100 person-years in 2009.^5^ With the increasing coverage of ART, the emergence of acquired drug resistance (ADR) is arising.^6^

The overall prevalence of ADR was estimated to be 44.7% with a region-specific pattern in China during 2001–17.^7^ A previous study reported an ADR prevalence of 53.3% in Shanghai during 2008–15.^8^ In this study, we characterized HIV-1 subtypes and the profile of drug resistance mutations (DRMs) in ART-treated PLWH in Shanghai during 2017–21.

## Methods

### Ethics

This study was approved by the Ethics Committee of Shanghai Public Health Clinical Center (SPHCC) (No. 2021-S051-01). All participants gave written informed consent.

### Study population

The study included 8669 PLWH who visited and/or received ART therapy in SPHCC during January 2017 to December 2021.

### HIV RNA extraction and pol gene amplification

Five millilitres of peripheral blood was collected from each involved PLWH. Plasma was separated by centrifugation. Viral RNA was extracted from plasma using the QIAamp Viral RNA Mini Kit (QIAGEN, Germany) according to the manufacturer protocol. The cDNA was synthesized using the PrimeScript^™^ 1st Strand cDNA Synthesis Kit (Takara Biomedical Technology Co., Ltd) with a specific primer according to the instructions. Partial HIV-1 *pol* gene (HXB2: 2147–3462), which includes the reverse transcriptase and protease coding regions, was amplified using a nested PCR as previously described.^9^ PCR products were tested with a 1% agarose gel and then sent for Sanger sequencing at BioSune Biotechnology Co. (Applied Biosystems, 3730xl).

### Phylogenetic analyses and determination of HIV-1 subtype

The reference sequences of HIV-1 subtypes/CRFs were downloaded from the HIV Sequence Database (https://www.hiv.lanl.gov/content/sequence/HIV/mainpage.html) on 12 May 2023 and aligned with obtained sequences using MAFFT v7.425. The maximum-likelihood tree was constructed with the general time reversible (GTR) model in IQ-TREE v.2.1.4 with 1000 bootstrap replications.

### Identification of DRMs

The DRMs and antiretroviral susceptibility of obtained sequences were analysed using the Stanford University HIV Drug Resistance Database (https://hivdb.stanford.edu/hivdb/by-patterns/). The degree of drug resistance was classified into five levels: susceptible, potential low-level resistance, low-level resistance, intermediate resistance and high-level resistance.

### Statistical analysis

Differences between groups were compared with the chi-squared test in SPSS 26.0. A *P* value less than 0.05 was considered to be significant.

### Sequence data

The sequences with DRMs in this study were deposited to GenBank under the accession numbers OR174998–OR176512.

## Results

### Characteristics of the participants

A total of 8669 PLWH, of whom 7952 (91.7%) were male and 717 (8.3%) female, were recruited in this study. The majority of them were young people with ages below 40 years (Table S1, available as Supplementary data at *JAC* Online). Ten HIV-1 subtypes/CRFs, including A, B, C, G, CRF01_AE, CRF07_BC, CRF08_BC, CRF51_01B, CRF55_01B and CRF68_01B, were identified. CRF01_AE (46.8%) and CRF07_BC (35.7%) are the top two HIV-1 genotypes circulating in Shanghai, followed by B (6.4%) and CRF55_01B (2.8%) (Table S1).

### Analysis of DRM sites

**Figure 1.**
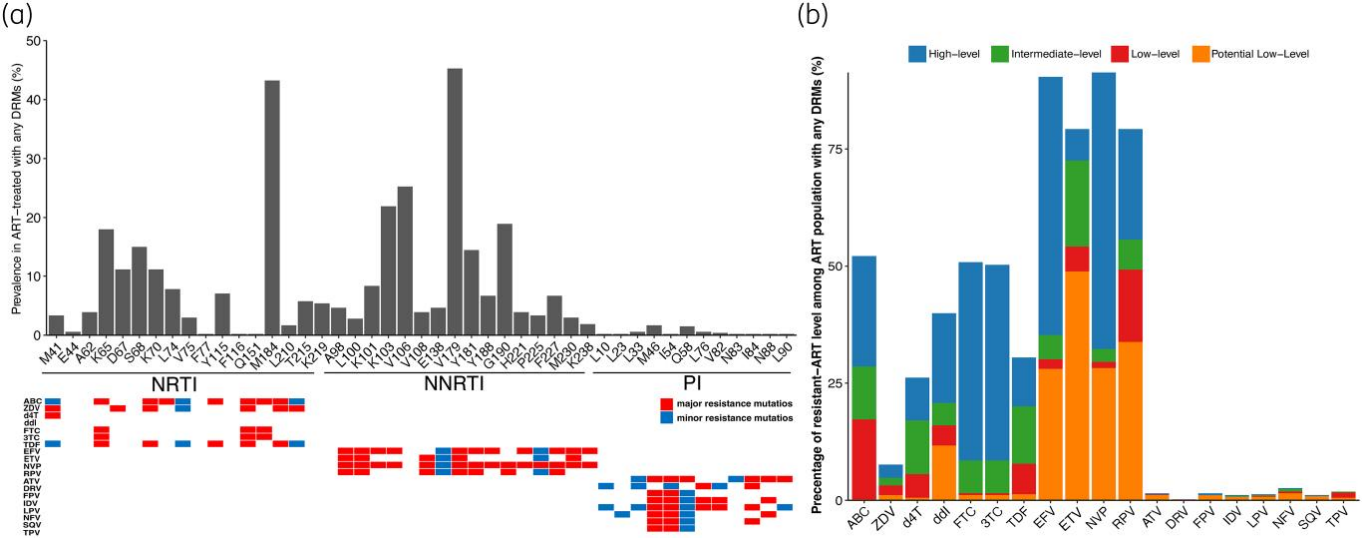
The prevalence of HIV-1 drug resistance mutations (a) and their resistance levels to various antiretroviral drugs (b) in ART-treated PLWH. The major and minor resistance mutations are highlighted in red and blue, respectively. ABC, abacavir; ZDV, zidovudine; d4T, stavudine; ddI, didanosine; FTC, emtricitabine; 3TC, lamivudine; TDF, tenofovir disoproxil fumarate; EFV, efavirenz; ETV, etravirine; NVP, nevirapine; RPV, rilpivirine; ATV, atazanavir; DRV, darunavir; FPV, fosamprenavir; IDV, indinavir; LPV, lopinavir; NFV, nelfinavir; SQV, saquinavir; TPV, tipranavir. This figure appears in colour in the online version of *JAC* and in black and white in the print version of *JAC*.

A total of 1559 (18.0%) sequences were found carrying DRMs. Of these, 539 (34.6%) sequences were obtained from 811 ART-treated PLWH with virological failure. The total prevalence of ADR in Shanghai was 48% (389/811) during 2017–21 (Table S1). The ADR prevalence appeared to be slightly fluctuating (44.8%–67.1%) over years (Figure S1). Seventeen NRTI-associated, 16 NNRTI-associated and 12 PI-related mutation sites were identified (Figure 1a). The most common NRTI DRM was M184V/I/L (43.2%), followed by K65R/N (18.0%), S68G/N/R (15.0%), D67N/E/G//T (11.1%) and K70E/R/G/Q/N/T (11.1%), and the most common NNRTI DRM was V179D/E/T/L (45.3%), followed by K103N/R/S/T (21.9%), V106M/I/A (25.2%) and Y181C/I (14.4%) (Table 1). All PI-associated DRMs had very low frequency (<2.0%).

**Table 1.** HIV DRM profiles in ART-treated PLWH by subtype

Mutation proportion % (*N*)	Sites	B (*n* = 56)	CRF01_AE (*n* = 309)	CRF07_BC (*n* = 86)	CRF55_01B (*n* = 32)	CRF08_BC (*n* = 17)	other (*n* = 39)	Total (*n* = 539)	χ^2^	*P*
Resistance to NRTIs	M41L	3.6 (2)	2.9 (9)	4.7 (4)		5.9 (1)	5.1 (2)	3.3 (18)	3.096	0.617
	E44A/D		0.6 (2)				2.6 (1)	0.6 (3)	4.184	0.552
	A62V	3.6 (2)	4.9 (15)	1.2 (1)			7.7 (3)	3.9 (21)	4.628	0.375
	K65R/N	14.3 (8)	22.3 (69)	10.5 (9)	9.4 (3)		20.5 (8)	18.0 (97)	13.263	**0.018**
	D67N/E/G/S	8.9 (5)	13.3 (41)	9.3 (8)	3.1 (1)	5.9 (1)	10.3 (4)	11.1 (60)	3.734	0.577
	S68G/N/R	8.9 (5)	21.0 (65)	2.3 (2)	6.3 (2)		17.9 (7)	15.0 (81)	28.977	**0**
	K70/E/R/G/Q/N/T	19.6 (11)	12.9 (40)	8.1 (7)		5.9 (1)	2.6 (1)	11.1 (60)	13.174	**0**.**016**
	L74I/V	5.4 (3)	10.0 (31)	5.8 (5)			12.8 (5)	8.2 (44)	7.243	0.17
	V75I/M	1.8 (1)	3.9 (12)	1.2 (1)	3.1 (1)		2.6 (1)	3.0 (16)	1.727	0.871
	F77L		0.3 (1)					0.2 (1)	5.495	1
	Y115F	5.4 (3)	8.7 (27)	4.7 (4)	3.1 (1)		7.7 (3)	7.1 (38)	3.047	0.671
	F116Y		0.3 (1)					0.2 (1)	5.495	1
	Q151M		0.3 (1)					0.2 (1)	5.495	1
	M184V/I/L	33.9 (19)	51.5 (159)	31.4 (27)	21.9 (7)	17.6 (3)	46.2 (18)	43.2 (233)	26.165	**0**
	L210W		1.6 (5)	2.3 (2)			5.1 (2)	1.7 (9)	3.631	0.473
	T215Y/F/A/D/S/I/L/V	7.1 (4)	7.1 (22)	3.5 (3)			5.1 (2)	5.8 (31)	3.809	0.532
	K219E/N/Q/R	5.4 (3)	6.8 (21)	3.5 (3)	3.1 (1)		2.6 (1)	5.4 (29)	2.129	0.825
Resistance to NNRTIs	A98G	1.8 (1)	3.6 (11)	3.5 (3)	12.5 (4)		15.4 (6)	4.6 (25)	12.997	**0**.**012**
	L100I	3.6 (2)	2.6 (8)	2.3 (2)	3.1 (1)		5.1 (2)	2.8 (15)	1.898	0.844
	K101E/H/P	7.1 (4)	10.7 (33)	5.8 (5)			7.7 (3)	8.3 (45)	6.476	0.229
	K103N/R/S/T	35.7 (20)	20.1 (62)	24.4 (21)	15.6 (5)	11.8 (2)	20.5 (8)	21.9 (118)	8.156	0.142
	V106M/I/A	23.2 (13)	31.1 (96)	18.6 (16)	3.1 (1)	11.8 (2)	20.5 (8)	25.2 (136)	19.442	**0**.**001**
	V108I	1.8 (1)	5.5 (17)	2.3 (2)		5.9 (1)		3.9 (21)	4.816	0.351
	E138A/G/K/Q	1.8 (1)	3.9 (12)	7.0 (6)	6.3 (2)	11.8 (2)	5.1 (2)	4.6 (25)	5.229	0.316
	V179D/E/L/T	39.3 (22)	44.3 (137)	38.4 (33)	100 (32)	47.1 (8)	30.8 (12)	45.3 (244)	44.589	**0**
	Y181C/I/V	12.5 (7)	15.5 (48)	9.3 (8)	18.8 (6)		23.1 (9)	14.5 (78)	7.968	0.145
	Y188C/I	7.1 (4)	7.1 (22)	3.5 (3)	9.4 (3)		10.3 (4)	6.7 (36)	3.684	0.566
	G190A/S/T/C/D/E/Q	16.1 (9)	24.6 (76)	9.3 (8)		11.8 (2)	17.9 (7)	18.9 (102)	22.331	**0**
	H221Y	7.1 (4)	4.5 (14)				7.7 (3)	3.9 (21)	8.546	0.078
	P225H	5.4 (3)	2.9 (9)	4.7 (4)		5.9 (1)	2.6 (1)	3.3 (18)	3.204	0.59
	F227L/I		8.4 (26)	9.3 (8)			5.1 (2)	6.7 (36)	9.779	0.057
	M230L		4.2 (13)	3.5 (3)				3.0 (16)	3.616	0.492
	K238T	1.8 (1)	2.9 (9)					1.9 (10)	2.96	0.613
Resistance to PIs	L10F		0.3 (1)					0.2 (1)	5.495	1
	L23LIV			1.2 (1)				0.2 (1)	8.053	0.427
	L33F		0.6 (2)				2.6 (1)	0.6 (3)	4.184	0.552
	M46I/L/V	1.8 (1)	1.9 (6)		3.1 (1)		2.6 (1)	1.7 (9)	3.095	0.587
	I54L		0.3 (1)					0.2 (1)	5.495	1
	Q58E		0.3 (1)	8.1 (7)				1.5 (8)	17.097	**0**.**001**
	L76V		0.6 (2)				2.6 (1)	0.6 (3)	4.184	0.552
	V82A/G		0.3 (1)	1.2 (1)				0.4 (2)	4.32	0.672
	N83D		0.3 (1)					0.2 (1)	5.495	1
	I84V		0.3 (1)					0.2 (1)	5.495	1
	N88T			1.2 (1)				0.2 (1)	8.053	0.427
	L90M						2.6 (1)	0.2 (1)	9.635	0.163

Bold type indicates statistical significance.

### The drug sensitivity of sequences with DRMs

The drug resistance levels of sequences with any DRMs were evaluated for 19 drugs, including seven NRTI, four NNRTI and eight PI drugs (Figure 1b). The prevalence of drug resistance to NRTIs, NNRTIs and PIs were 39.2%, 62.0% and 2.8%, respectively (Figure S2). The percentage of NRTI/NNRTI dual-class resistance was observed at 36.3%. The percentages of high-level resistance to efavirenz and nevirapine were 55.1% and 59.0% (Figure 1b). In the NRTI group, abacavir, emtricitabine and lamivudine resistance was more common than for other drugs (e.g. didanosine, tenofovir, stavudine and zidovudine).

### Characteristics of DRMs in different HIV subtypes

DRMs could be found in each of 10 HIV-1 subtypes/CRFs (Table 1). CRF01_AE (57.3%) was the most common subtype carrying any DRMs, followed by CRF07_BC (16.0%) and B (10.4%) (Table S1). Seventeen NRTI-associated, 16 NNRTI-associated and 12 PI-associated DRMs were identified (Table 1). Of these, four NRTI-associated (K65R/N, S68G/N/R, K70/E/R/G/Q/N/T and M184V/I/L), four NNRTI-associated (A98G, V106M/I/A, V179D/E/L/T and G190A/S/T/C/D/E/Q) and one PI-associated (Q58E) showed significantly different prevalence among different subtypes/CRFs (*P* < 0.05). All 17 NRTI-associated DRMs were found in CRF01_AE, followed by 13 in CRF07_BC and 12 in subtype B. The top three NRTI-associated DRMs were M184V/I/L (43.2%), K65R/N (18.0%) and S68G/N/R (15.0%), and all of them had the highest prevalence in CRF01_AE (51.5%, 22.3%, and 21.0%, respectively). All 16 NNRTI-associated DRMs were also found in CRF01_AE, and 14 were found in subtype B and CRF07_BC. The top four NNRTI-associated DRMs were V179D/E/T/L (45.3%), V106M/I/A (25.2%), K103N/R/S/T (21.9%) and G190A/S/T/C/D/E/Q (18.9%), all which had the highest prevalence in CRF01_AE (20.1%–44.3%) except K103N/R/S/T having the highest prevalence in subtype B (35.7%). Of PI-associated DRMs, nine and four were found in CRF01_AE and CRF07_BC, respectively, and only Q58E showed a relatively high prevalence (8.1%) in CRF07_BC.

## Discussion

In this study we conducted a retrospective epidemiological investigation to clarify the prevalence and characteristics of HIV-1 drug resistance in Shanghai from 2017 to 2021. ADR prevalence was estimated to be 48%. The first-line ART regimen containing tenofovir, efavirenz and lamivudine was administered at the time of virological failure. NRTI-associated mutations M184V/I/L, S68G/N/R and K65R/N, and NNRTI-associated mutations V179D/E/T/L, V106M/I/A and K103N/R/S/T were the most common observed DRMs in ART-treated PLWH, and showed significantly different prevalence among subtypes/CRFs. The results indicated that the predominant DRMs have evolved from one NRTI-associated (M184V/I) and three NNRTI-associated (K103N/S, Y181C/I and G190A/S) DRMs before 2017 to the current three NRTI-associated (M184V/I/L, S68G/N/R and K65R/N) and three NNRTI-associated (V179D/E/T/L, V106M/I/A and K103N/R/S/T) DRMs, respectively,^10^ which might be associated with the revision of first-line ART regimens.

Currently, eight free drugs, including four NRTI drugs (zidovudine, abacavir, tenofovir and lamivudine), three NNRTI drugs (efavirenz, nevirapine and rilpivirine) and one PI drug (lopinavir), are available in Shanghai. The regimen composed of tenofovir, lamivudine and efavirenz is the most commonly used for free first-line therapy. M184V/I/L can cause high-level resistance to lamivudine and low or intermediate resistance to abacavir. K65R confers intermediate reductions in susceptibility to tenofovir and abacavir. Co-presence of M184V/I with K65R, as well as L74V or Y115F mutations increases resistance to abacavir, which explains the highest level of drug resistance to abacavir. S68G is a natural polymorphism and has often occurred in conjunction with K65R, which improves virus replication.^11^ DRMs K103N, V106M and G190A cause high-level resistance to nevirapine and efavirenz.^12^ V179D/E, a polymorphic accessory NNRTI-selected mutation with low-level resistance, was found to have the highest prevalence. A rising trend of co-occurrence of both V179D/E and E138G in CRF55_01B was previously observed in ART-treated PLWH.^13^ E138G can result in low-level resistance to rilpivirine and doravirine. Doravirine is a new NNRTI, which has not been widely use in China.^14^ A rising prevalence of E138G is notable since it increases the resistance risk to rilpivirine. Very low prevalence of PI-associated DRMs encourages an inclusion of PI drug (e.g. lopinavir) in current ART regimens.

Ten HIV-1 subtypes/CRFs were identified, showing a complex HIV-1 genetic diversity in Shanghai. The predominant HIV-1 subtype was CRF01_AE (46.8%), followed by CRF07_BC (35.7%), B (6.4%) and CRF55_01B (2.8%). The prevalence and distribution of DRMs appeared to be significantly different among main subtypes/CRFs. In view of the highest prevalence of CRF01_AE and CRF07_BC in Shanghai, ongoing monitoring of HIV-1 drug resistance in patients infected with CRF01_AE or CRF07_BC should be highly encouraged. On the other hand, several high-prevalence DRMs (e.g. NNRTI-associated DRMs: M184V/I/L, S68G/N/R and K65R/N; and NNRTI-associated DRMs: V179D/E/T/L, V106M/I/A and K103N/R/S/T) were shared by these main subtypes/CRFs. These DRMs should be closely monitored.

In summary, we revealed ADR prevalence of 48% in Shanghai during 2017–21, and identified 10 HIV-1 subtypes/CRFs. Main DRMs were found to have significantly different prevalence among different subtypes/CRFs.

## Supplementary Material

dkad370_Supplementary_Data

## Supplementary data

Figures S1 and S2 and Table S1 are available as Supplementary data at *JAC* Online.
