# Supplementary material for: The profile of HIV-1 drug resistance in Shanghai, China: a retrospective study from 2017 to 2021
Source: J Antimicrob Chemother. 2024 Feb 1;79(3):526–30. doi: 10.1093/jac/dkad370 (PMC10904715; doi:10.1093/jac/dkad370)
Supplement: dkad370_Supplementary_Data [file dkad370_supplementary_data.zip › Supplementary_HIV-1114.docx]

Supplementary Material

***for***

**The profile of HIV-1 drug resistance in Shanghai, China: A Retrospective Study from 2017 to 2021**

Min ZHANG^1, †^, Yingying MA^1, †^, Gang WANG^1, †^, Zhenyan WANG^1^, Qianying WANG^1^, Xin LI^1^, Feng LIN^1^, Jianping QIU^1^, Daihong CHEN^1^, Yinzhong SHEN^1^, Chiyu ZHANG^1, ‡^, Hongzhou LU^1, 2, ‡ *^

^1^ Shanghai Clinical Research Center for Infectious Disease (HIV/AIDS), Shanghai Public Health Clinical Center, Fudan University, Shanghai, 201508, China

^2^ The Third People’s Hospital of Shenzhen, Second Hospital Affiliated to Southern University of Science and Technology, Shenzhen 518112, Guangdong, China

^†^ These authors contributed equally to this study.

^‡^ Both senior authors contributed equally to this study.

^*^ Corresponding author. E-mail: [luhongzhou@fudan.edu.cn](mailto:luhongzhou@fudan.edu.cn)


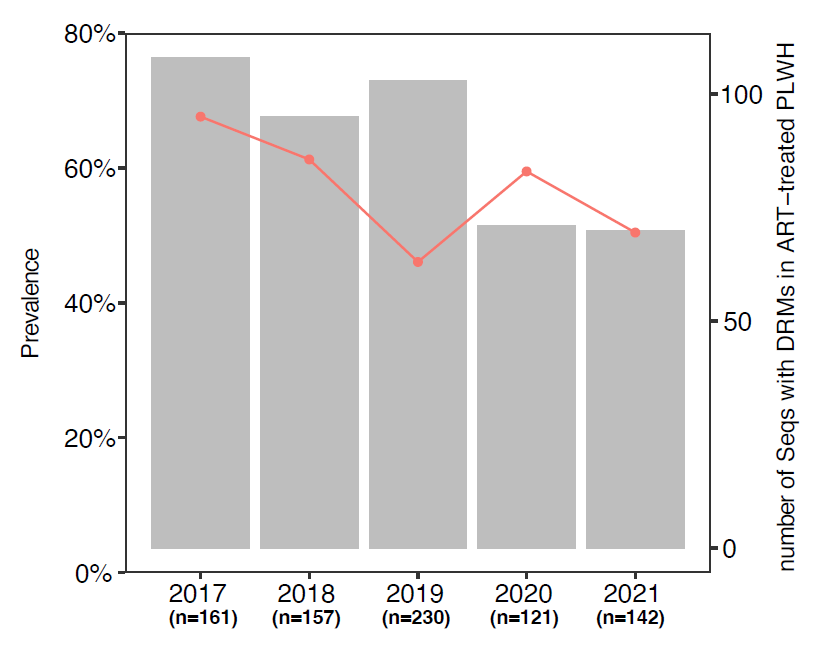


**Fig. S1.** Number of Seqs with DRMs and ADR prevalence by years (2017-2021).

**Figure S2.** Prevalence of HIV drug resistance in ART-treated PLWH by drug class.
