# Supplementary material for: The profile of HIV-1 drug resistance in Shanghai, China: a retrospective study from 2017 to 2021
Source: J Antimicrob Chemother. 2024 Feb 1;79(3):526–30. doi: 10.1093/jac/dkad370 (PMC10904715; doi:10.1093/jac/dkad370)
Supplement: dkad370_Supplementary_Data [file dkad370_supplementary_data.zip › updated table S1 .pdf]

Table S1. The demographic information of PLWH in Shanghai, China.

Variable	Total patients, n (%)	DRM, n (%)	ART-treated with DRM, n (%)
Gender			
male	7952 (91.7)	1453 (93.2)	501 (92.9)
female	717 (8.3)	106 (6.8)	38 (7.1)
Age (years)			
5-30	3301 (38.1)	682 (43.7)	274 (50.8)
31-40	2410 (27.8)	389 (25.0)	117 (21.7)
41-45	584 (6.7)	108 (6.9)	32 (5.9)
46-55	1081 (12.5)	194 (12.4)	52 (9.6)
56-65	901 (10.4)	131 (8.4)	38 (7.1)
>65	392 (4.5)	55 (3.5)	26 (4.8)
Subtype			
B	553 (6.4)	134 (8.6)	56 (10.4)
CRF01_AE	4056 (46.8)	825 (52.9)	309 (57.3)
CRF07_BC	3094 (35.7)	218 (14.0)	86 (16.0)
CRF55_01B	245 (2.8)	215 (13.8)	32 (5.9)
CRF08_BC	207 (2.4)	48 (3.1)	17 (3.2)
other	514 (5.9)	119 (7.6)	39 (7.2)
ART-treated (including individuals with potential low-level resistance)			
ART-treated	811 (9.4)	539 (66.4)	
ADR (without individuals with potential low-level resistance)			
ADR	811 (9.4)	389 (48.0)	

ADR: acquired drug resistance
